# The Book World of Medicine and Science

**Published:** 1906-05-19

**Authors:** 


### The Book World of Medicine and Science.

Aids to Surgical Diagnosis. By H. W. Carson, F.R.C.S. (London : Baillière, Tindall, and Cox. 1906. Pp. 140. Price 3s. 6d.)

The author of this little book tells us in his Preface that it is "intended for the use of students and practitioners who may desire to obtain in a small compass the main points in the diagnosis of the more common surgical diseases." That he has succeeded in including the diagnosis of all the more important surgical conditions within the space at his disposal is rather a matter for marvel than congratulation. The dismissal of the most intricate surgical questions in a few lines renders the book useless to the beginner, to whom the morbid conditions referred to are unfamiliar, while to the student of greater experience something more than an outline of the facts is generally necessary. The use of such books in the preparation for examinations is to be deprecated, as they are apt to take the place not merely of the student's personal observation, which forms the only basis of a true clinical knowledge of disease, but also the wider reading which is its proper complement. By the dogmatic utterances necessitated by their condensed form such books tend to produce clinical pictures which often serve as pitfalls rather than aids to diagnosis.

Nutrition and Dysentery. By U. N. Mukerji, M.D.Edin., M.R.C.S.Eng. (Calcutta : S. K. Lahiri and Company; London : Kegan, Paul, and Trench. Pp. 286. Price 3s. 6d. net.)

This suggestive *brochure* is not only an interesting contribution to the physiology of nutrition and the therapeutics of dysentery, but is also a valuable work for its side-lights on medico-sociological questions relating to native customs and the life of Anglo-Indians. Dr. Mukerji, as medical officer of a Bengal gaol, has enjoyed exceptional opportunities for the systematic study of his subject. He shows how the lack of fish and customary inunction of oil may prove inimical to the health of prisoners. The hepatic and alimentary disturbances ensuing from interference with native habits is apparently of considerable importance in leading to dysentery. For the treatment of this oftentimes serious malady the author speaks highly of calomel. Dr. Mukerji compares the dietary of a British officer in India with a Bengali agricultural labourer and an Oxford undergraduate, and has no difficulty in showing that the unphysiological extravagance of the first is a serious predisposing factor in the ætiology of disease. Much of the book is taken up with the records of urea estimation of prisoners under various dietetic and other conditions, and throughout there is evidence not only of a well-directed industry, but a scientific spirit energised by originality of thought and keen humanitarian interest.

